# Amino Acid Composition of Milk from Cow, Sheep and Goat Raised in Ailano and Valle Agricola, Two Localities of ‘Alto Casertano’ (Campania Region)

**DOI:** 10.3390/foods10102431

**Published:** 2021-10-13

**Authors:** Nicola Landi, Sara Ragucci, Antimo Di Maro

**Affiliations:** Department of Environmental, Biological and Pharmaceutical Sciences and Technologies (DiSTABiF), University of Campania ‘Luigi Vanvitelli’, Via Vivaldi 43, I-81100 Caserta, Italy; nicola.landi@unicampania.it (N.L.); sara.ragucci@unicampania.it (S.R.)

**Keywords:** amino acid profile, milk quality, raw proteins, taurine

## Abstract

Cow, sheep and goat raw milk raised in Ailano and Valle Agricola territories (‘Alto Casertano’, Italy) were characterized (raw proteins, free and total amino acids content) to assess milk quality. Raw milk with the highest total protein content is sheep milk followed by goat and cow milk from both localities. Total amino acid content in cow, goat and sheep raw milk is 4.58, 4.81 and 6.62 g per 100 g, respectively, in which the most abundant amino acid is glutamic acid (~20.36 g per 100 g of proteins). Vice versa, the free amino acids content characteristic profiles are different for each species. In particular, the most abundant free amino acid in cow, sheep and goat raw milk is glutamic acid (9.07 mg per 100 g), tyrosine (4.72 mg per 100 g) and glycine (4.54 mg per 100 g), respectively. In addition, goat raw milk is a source of taurine (14.92 mg per 100 g), retrieved in low amount in cow (1.38 mg per 100 g) and sheep (2.10 mg per 100 g) raw milk. Overall, raw milk from ‘Alto Casertano’ show a high total protein content and are a good source of essential amino acids.

## 1. Introduction

Milk is a fluid secreted by the female of all mammalian species necessary for the nutritional requirements of the neonate [1]. Milk is an emulsion of oil in water (~88%), containing bioactive proteins, lipids and saccharides, as well as main biologically active substances such as antibodies, enzymes, antimicrobial peptides, oligosaccharides and hormones [2]. The main role of milk is to provide energy (lipids and lactose), essential amino acids, fatty acids, vitamins, inorganic elements and water [3,4]. Considering the content of these substances, after childhood humans continue to consume milk from various species such as cattle, goats, sheep, water buffalo, camel, donkey and horse. Moreover, the different technological treatments or transformations of raw milk make this food or its derivatives (e.g., cheese, cream, butter, yogurt and kefir) always available [4].

The protein content of raw milk differs among the species intended for human consumption; indeed, the sheep raw milk has the higher protein content (5.5%) followed by water buffalo, camel, cattle, goat, horse and donkey raw milks (4.4–5.1%; 3.9%; 3.4%; 2.9%; 2.5% and 2.0%, respectively) [5]. In addition, the major contribution in terms of milk nutritional value, consists of caseins (α_S1_, α_S2_, β and κ) and whey protein, rich in essential and non-essential amino acids, having highest biological value, good digestibility, rapid absorption and utilization [6]. For example, in cow, sheep and goat, approximately 80% of the proteins present in raw milk consists of four proteins named caseins (α_S1_, α_S2_, β and κ-caseins) [7]. On the other hand, the percentage of each casein changes according to the species. In particular, the total casein content in cow raw milk is ~80% of total proteins, where α_S1_, α_S2_, β and κ-casein represent 37%, 7%, 42% and 9%, respectively [8]. Moreover, the total casein content in sheep raw milk is 85% of total proteins, where α_S1_, α_S2_, β and κ-casein represent 6.7%, 22.8%, 61.6% and 8.9%, respectively, as reported by Balthazar et al., 2017 [9]. The same authors reported that the total casein content in goat raw milk is 65% of total proteins, where α_S1_, β and κ-casein represent 5.6%, 54.8% and 20.4%, respectively, while α_S2_ (generally 19.2%) is highly dependent on the genotype [9].

The remaining 20% of milk proteins includes major whey proteins β-lactoglobulin and α-lactalbumin as well as other protein constituents: immunoglobulins, serum proteins, milk fat globule proteins, transferrin, lactoferrin, β2-microglobulin, several enzymes, peptides and proteolytic products [10]. On the other hand, the protein content of raw milk and their amino acid profiles from various species show great variations both during infant’s growth and among species, considering the different growth rate and energy requirements [11]. In addition, genetic, physiological and nutritional factors, as well as environmental conditions, play a great role in these differences [6,12,13].

At the same time, it is known that consumers prefer milk and dairy products, which have favorable sensory qualities, depending on the influence of territorial dietary factors. These differences improve local production of milk and support the conservation of regional resources and related territories [7]. Therefore, acquiring information on the amino acid profile of raw milk proteins from different species is important for an adequate protein uptake and consumption [14] and to add data on the quality of these milk samples.

**Figure 1:**
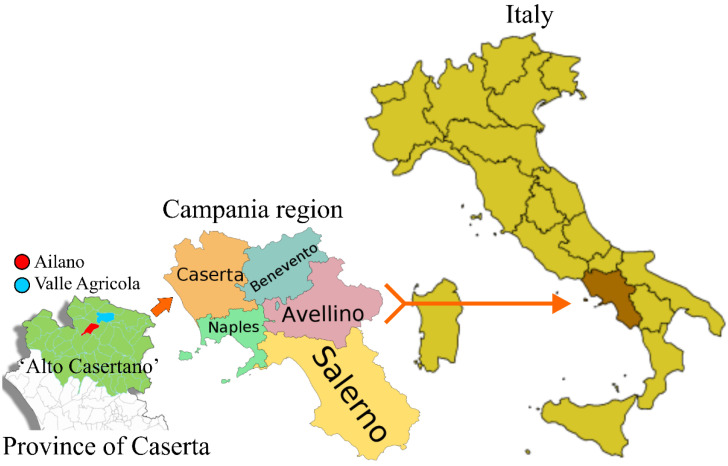
Localities (territory areas) of Ailano and Valle Agricola in which raw milks were sampled are highlighted in red and blue, respectively.

In this scenario, the present work describes the total protein content (caseins and whey proteins) and the total and free amino acid profiles of raw milk from three different local species found in the mountain community of ‘Alto Casertano’ (Campania region, Italy). In particular, the study was carried out on cow, sheep and goat lactating breeds raised in the territory of Ailano (41°23′ N 14°12′ E; elevation: 260 m) and Valle Agricola (41°25′ N 14°15′ E; elevation: 691 m), Figure 1.

These territories have a rich tradition regarding the breeding of the three species object of this work. Indeed, today there are still small farms located in Ailano and Valle Agricola, where the animals are reared in a semi-wild state. In particular, this type of breeding consists of keeping the animals in the stable during winter and grazing during summer. Furthermore, raw milk produced during summer period is used for the preparation of local dairy products such as ‘caciocavallo’, ‘scamorza’, fresh and seasoned ‘ricotta’, as well as many other kinds of cheese, considered typical products of these territories. Therefore, having more information on the total protein content and the total and free amino acid profile of the raw milk produced in Ailano and Valle Agricola territories (‘Alto Casertano’) could be of interest for the enhancement of raw milk that is also the starting material for the obtainment of typical dairy products.

## 2. Materials and Methods

### 2.1. Chemicals and Reagents

The sources of chemicals used in this work were obtained from Sigma-Aldrich (St. Louis, MO, USA). Chemicals, solvents and reagents for the Kjeldahl method were purchased from Sigma-Aldrich and Carlo Erba Reagents (Milan, Italy). Buffers and reagents for automated amino acid analysis were provided from Biochrom (Cambridge, UK).

### 2.2. Milk Samples and Samples Analyzed

**Scheme 1:**
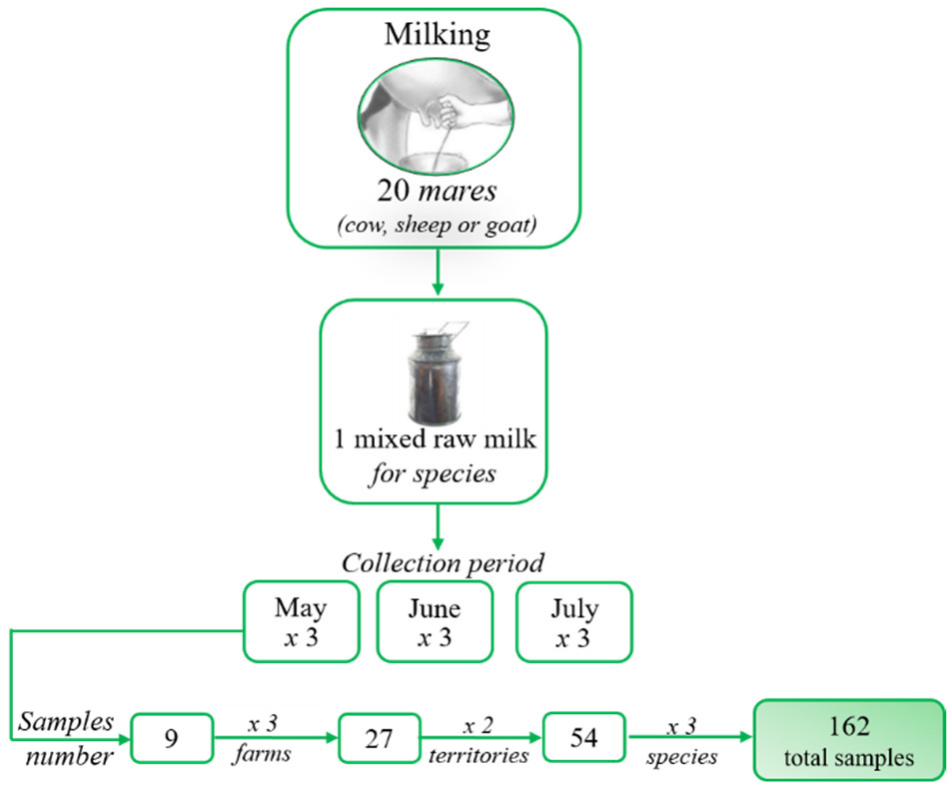
Schematic representation of raw milk samples collection analyzed.

Raw milk samples from cow, sheep and goat (crossbred) were collected from three different farms located in Ailano and Valle Agricola (Caserta, Italy). Each sample was obtained by mixing the raw milk obtained from 20 mares of the crossbred for each species that foaled during the period from February to April. The sampling was made monthly from May to July, repeating the procedure in three random days for each month. Moreover, during the sampling period, the mares were reared on pasture without any form of concentrated integration. The collection procedure followed the norms of good milking practices: the breasts were washed with water and the first three milk jets were discarded in a black bottom mug to verify the presence of lumps. Overall, from each farm, 9 samples of cow, sheep and goat raw milk were collected (27 samples for species from Ailano and Valle Agricola, respectively) and analyzed in triplicate (Scheme 1).

At the end of milking, homogeneous raw milk samples were collected and stored in polypropylene bottles (Fal-con, Becton Drive, Franklin Lakes, NJ, USA) at 4 °C during transport. Aliquots of samples were stored at −80 °C until use. Therefore, the results reported in all Tables represent the average values for each raw milk.

### 2.3. Nitrogen Determination

Total protein (TP), non-protein nitrogen (NPN) and protein nitrogen (PN) were determined using the Kjeldahl method [15]. For the analysis of TP, about 5.0 g of raw milk were mineralized using a Mineral Six digester (VWR International PBI, Milan, Italy) and then an Auto Disteam semi-automatic distilling unit (VWR International PBI) was used for determining the nitrogen percentage. Finally, TP was estimated using a nitrogen factor of 6.38.

Subsequently, for the analysis of non-protein nitrogen (NPN; urea, peptides, ammonium, free amino acids and other minor nitrogen containing compounds) and protein nitrogen (PN; caseins and whey proteins), about 5.0 g of raw milk were centrifuged (3000× *g*) in JA.25–50 rotor at 4 °C for 20 min in order to remove fat. After filtering on N. 3 Whatman paper, an equal volume of 24% TCA was added to precipitate proteins. After centrifugation at 3000× *g* for 10 min at 4 °C, protein precipitate was separated from the supernatant filtering on N. 3 Whatman paper. Finally, the precipitated proteins and the supernatant were obtained, containing, respectively, PN and NPN, were analyzed using the Kjeldahl method [15] to estimate the nitrogen percentage.

### 2.4. Amino Acid Analysis

For the analysis of free amino acid composition, three aliquots of 200 µL of different raw milk types were precipitated, using 99% cold ethanol (800 µL) in the presence of 200 nmol of nor-Leucine (nor-Leu) as an internal standard, homogenized with a Teflon pestle and centrifuged at 14,000× *g* at 4 °C. The supernatants were lyophilized, treated with 3% sulfosalicylic acid (500 μL) to precipitate any protein fraction still present and centrifuged again [16,17]. Thus, the supernatants obtained were analyzed.

For the analysis of total amino acid content (free plus protein), 200 µL of different raw milk types were freeze-dried and then hydrolyzed with 200 µL of 6 N HCl containing 0.02% phenol and nor-Leu (50 nmol) as an internal standard at 110 °C for 20 h [18]. Following hydrolysis, HCl was removed under vacuum and the samples were re-suspended in 0.5 mL of 0.2 M Li-citrate buffer (pH 2.2).

Aliquots of hydrolyzed and non-hydrolyzed samples were directly analyzed on a Biochrom30 amino acid analyzer (Biochrom, Cambridge, UK) equipped with a post-column ninhydrin derivatization system [19]. Chemicals and experimental conditions were as suggested by the manufacturer.

### 2.5. Cysteine Oxidation with Performic Acid

Protein milk samples were subjected to oxidation with performic acid. Samples were essentially treated as previously reported [20]. Briefly, 200 μL of different raw milk types were freeze-dried and then hydrolyzed in a glass tube and 400 μL of performic acid were added. After incubation at 0 °C for 60 min, 200 μL of cold HBr were added. Samples were taken to dryness in a desiccator, rinsed with water and then the hydrolyzed samples were analyzed following the correct procedure.

### 2.6. Statistical Analysis

All the analyses were repeated three times and the data are expressed as mean ± standard deviation (SD). Data analysis was conducted using Excel Office 2016 (Microsoft Corporation, Redmond, WA, USA). The Bonferroni post-test was used to determine significant differences. The test was performed using a *p* < 0.05 using GraphPad Prism 5.0 software (GraphPad Software Inc., San Diego, CA, USA).

## 3. Results and Discussion

### 3.1. Total Proteins and Non-Protein Nitrogen in Raw Milk

**Figure 2:**
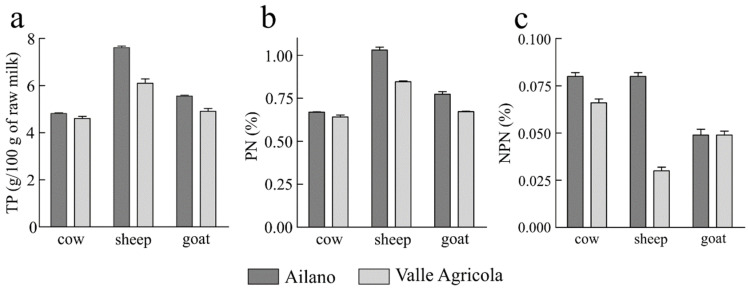
(**a**) Total protein content (TP), (**b**) protein nitrogen (PN) and (**c**) non-protein nitrogen (NPN) content of cow, goat and sheep milk from ‘Ailano’ and ‘Valle Agricola’ territories.

Milk is generally considered an important source of proteins present in different content in cow, sheep and goat milk types (3.2, 6.2 and 3.4 g/100 g of raw milk, respectively). In this study, the average amount of total proteins (TP) as well as protein nitrogen percentage (PN) and non-protein nitrogen percentage (NPN) content of cow, sheep and goat raw milk from ‘Ailano’ and ‘Valle Agricola’ territories are shown in Figure 2.

In particular, the TP content (Figure 2a) in Ailano cow raw milk (4.81 g/100 g of milk) is similar to Valle Agricola cow raw milk (4.60 g/100 g of milk). The TP content of sheep and goat raw milk from ‘Ailano’ (7.61 and 5.55 g/100 g of milk, respectively) is higher than that of sheep and goat raw milk from ‘Valle Agricola’ (6.10 and 4.91 g/100 g of milk, respectively). In addition, PN (caseins and whey proteins) was similar in cow raw milk produced from animals raised in both ‘Valle Agricola’ and ‘Ailano’ (0.669% and 0.641%, respectively), while PN was lower in both sheep and goat raw milk produced from animals raised in ‘Valle Agricola’, with respect to ‘Ailano’ (Figure 2b). Finally, the NPN content of cow and sheep raw milk from ‘Ailano’ (0.080%) is higher than of cow and sheep raw milk from ‘Valle Agricola’ (0.066% and 0.030%, respectively), while NPN content in goat raw milk is similar for both territories (~0.049%) (Figure 2c). The NPN fraction mainly consists of urea, peptides, ammonium, free amino acids and other minor nitrogen containing compounds [21].

### 3.2. Amino Acid Content of Cow Raw Milk from ‘Ailano’ and ‘Valle Agricola’

The total amino acid content (free plus protein) from hydrolyzed cow raw milk obtained by analyzing both ‘Ailano’ and ‘Valle Agricola’ samples and their average values are reported in Table 1. Moreover, no statistical differences were retrieved, except for histidine (His) and proline (Pro).

Subsequently, the average values were compared with those of cow raw milk reported by Claeys et al. [22], showing qualitative and quantitative differences. Considering the ‘Alto Casertano’ cow milk, Glx (glutamic acid + glutamine; 1.02 g/100 g) was the most abundant among total amino acids, followed by leucine (0.41 g/100 g), lysine (0.37 g/100 g), Asx (aspartic acid + asparagine; 0.35 g/100 g), proline (0.31 g/100 g) and serine (0.28 g/100 g), which represented about 60% of total amino acids. In addition, the amount of essential amino acids (His, Ile, Leu, Lys, Met, Phe, Thr, Val; -Trp (tryptophan is not included as it was not determined in the total hydrolyzed samples: see Table 1)) in ‘Alto Casertano’ cow raw milk was 1.96 g/100 g (~43% of total). The amount of methionine and cysteine in ‘Alto Casertano’ cow raw milk was 0.18 g/100 g (~4% of total), confirming the low level of sulfur amino acids found by Claeys et al. (the amount of sulfur amino acids was 0.10 g/100 g; ~3% of the total). On the other hand, cow milk contains a large amount of glutamic acid, which is 22% and 23% for ‘Alto Casertano’ and milk values reported by Claeys et al., respectively. Furthermore, leucine, lysine, Asx and serine content in ‘Alto Casertano’ cow raw milk were higher than the milk values reported by Claeys et al., while the proline content is the same (~0.31 g/100 g of milk).

In terms of free amino acids, the total amount in ‘Alto Casertano’ cow raw milk was 21.33 mg/100 g of milk (Table 2) and no statistically significant differences were retrieved, except for glutamic acid (Glu) and urea. Glutamic acid was by far the most abundant among the free protein amino acids (9.07 mg/100 g of milk). Furthermore, glycine (1.52 mg/100 g of milk), alanine (0.91 mg/100 g of milk), aspartic acid (0.70 mg/100 g of milk), lysine (0.41 mg/100 g of milk), proline (0.48 mg/100 g of milk) and arginine (0.34 mg/100 g of milk) were the most abundant free amino acids in ‘Alto Casertano’ cow raw milk. On the other hand, the amount of each other protein amino acid did not exceed 1.76 mg/100 g of product (~8% of free amino acids total content).

The analysis also evidenced the presence of ten non-protein amino acids (i.e., L-α-aminoadipic acid (AAAA); L-α-aminobutyric acid (ABAA); β-alanine (β-Ala); L-citrulline (Citr); ethanolamine (Ethan); L-ornithine (Orn); phosphorylethanolamine (Pea); phosphoserine (Phser); L-sarcosine (Sarc) and taurine (Taur)). The amount of these non-protein amino acids was 6.21 mg/100 g of milk (~29% of total). Finally, the analysis reveals that urea content in ‘Alto Casertano’ raw milk (43.87 mg/100 g of milk) represents about 10% of the non-protein nitrogen (NPN) fraction from ‘Alto Casertano’ cow raw milk, while free amino acids represent about 5% of NPN fraction.

### 3.3. Amino Acid Content of Sheep Raw Milk from ‘Ailano’ and ‘Valle Agricola’

Total amino acid content (free plus protein) from hydrolyzed sheep raw milk obtained by analyzing both ‘Ailano’ and ‘Valle Agricola’ samples and their average values were reported in Table 3. Moreover, no statistical differences were retrieved, except for some amino acids (i.d.: Ile, Leu, Lys, Phe, Thr, Val, Asx, Glx, Pro, Ser and Tyr). Subsequently, the average values were compared with those of sheep milk reported by Claeys et al. [22], showing qualitative and quantitative differences. In particular, Glx (glutamic acid + glutamine; 1.35 g/100 g) was the most abundant among the total amino acids in ‘Alto Casertano’ sheep raw milk, followed by proline (0.64 g/100 g), leucine (0.54 g/100 g), lysine (0.51 g/100 g), Asx (aspartic acid + asparagine; 0.50 g/100 g) and serine (0.39 g/100 g), which represent about 59% of the total amino acids.

In addition, the amount of essential amino acids (His, Ile, Leu, Lys, Met, Phe, Thr, Val; -Trp (tryptophan is not included as it was not determined in the total hydrolyzed samples: see Table 3)) in ‘Alto Casertano’ sheep raw milk was 2.72 g/100 g (~41% of total). The amount of methionine and cysteine in ‘Alto Casertano’ sheep raw milk was 0.26 g/100 g (~4.0% of total), confirming the low level of sulfur amino acids found by Claeys et al. (the amount of sulfur amino acids was 0.20 g/100 g; ~3% of the total).

On the other hand, sheep raw milk contains a large amount of glutamic acid, which is 20% and 17% for ‘Alto Casertano’ and milk values reported by Claeys et al., respectively. Furthermore, proline, Asx, threonine and phenylalanine content in ‘Alto Casertano’ sheep raw milk were higher than in milk reported by Claeys et al., while the other amino acids were present in lower quantities in ‘Alto Casertano’ sheep raw milk, compared to the information reported by Claeys et al. [22].

In terms of free amino acids, the total amount in ‘Alto Casertano’ sheep raw milk was 21.86 mg/100 g of milk (Table 4) and no statistically significant differences were found, except for glutamic acid (Glu), taurine (Taur), tyrosine (Tyr) and urea. Tyrosine was by far the most abundant among free protein amino acids (4.72 mg/100 g of milk), followed by Glu (glutamic acid; 2.98 mg/100 g of milk), glycine (1.12 mg/100 g of milk), asparagine (0.77 mg/100 g of milk) phenylalanine (0.73 mg/100 g of milk), arginine (0.71 mg/100 g of milk) and alanine (0.60 mg/100 g of milk).

On the other hand, the amount of each other protein amino acid did not exceed 2.8 mg/100 g of product (~13% of free amino acids total content). The analysis also revealed the presence of twelve non-protein amino acids (i.e., L-α-aminoadipic acid (AAAA); β-alanine (β-Ala); L-carnitine (Car); L-citrulline (Citr); ethanolamine (Ethan); 1-Methylhistidine (1-Mhis); 3-Methylhistidine (3-Mis); L-ornithine (Orn); phosphorylethanolamine (Pea); phosphoserine (Phser); L-sarcosine (Sarc); taurine (Taur)). The amount of these non-protein amino acids was 7.47 mg/100 g of milk (~34% of total). Finally, the analysis reveals that urea content in ‘Alto Casertano’ raw milk (59.60 mg/100 g of milk) represents about 17% of the NPN fraction from ‘Alto Casertano’ sheep raw milk, while free amino acids represent about 6% of the NPN fraction.

### 3.4. Amino Acid Content of Goat Raw Milk from ‘Ailano’ and ‘Valle Agricola’

The total amino acid content (free plus protein) of hydrolyzed goat raw milk obtained by analyzing both ‘Ailano’ and ‘Valle Agricola’ samples and their average values are reported in Table 5; moreover, no statistically significant differences were found, except for Glx (glutamic acid + glutamine). Subsequently, the average values were compared with those of the goat milk reported by Claeys et al. [22], showing qualitative and quantitative differences. In particular, Glx (glutamic acid + glutamine; 1.03 g/100 g) was the most abundant among the total amino acids in ‘Alto Casertano’ goat row milk, followed by proline (0.47 g/100 g), leucine (0.43 g/100 g), lysine (0.37 g/100 g) and Asx (aspartic acid + asparagine; 0.34 g/100 g), which represent about 55% of total amino acids. In addition, the amount of essential amino acids (His, Ile, Leu, Lys, Met, Phe, Thr, Val, -Trp (tryptophan is not included as it was not determined in the total hydrolysed samples: see Table 5)) in ‘Alto Casertano’ goat raw milk was 2.08 g/100 g (~43% of total). The amount of methionine and cysteine in ‘Alto Casertano’ goat raw milk was 0.19 g/100 g (~4.0% of total), confirming the low level of sulfur amino acids found by Claeys et al. [22] (the amount of sulfur amino acids was 0.13 g/100 g; ~4% of the total).

On the other hand, goat raw milk contains a large amount of glutamic acid, which is 21% and 18% for ‘Alto Casertano’ and the milk values reported by Claeys et al., respectively. Furthermore, the proline, Asx, threonine, leucine lysine and phenylalanine content in ‘Alto Casertano’ goat raw milk were higher than the milk values reported by Claeys et al., while the other amino acids were present in lower quantities in ‘Alto Casertano’ goat raw milk compared to in the milk values reported by Claeys et al. [22].

In terms of free amino acids, the total amount in ‘Alto Casertano’ goat raw milk was 46.15 mg/100 g of milk (Table 6), and no statistically significant differences were found except for taurine (Taur) and urea. Glycine was by far the most abundant among free protein amino acids (4.54 mg/100 g of milk; about 10% of total), followed by glutamic acid (4.12 mg/100 g), asparagine (2.75 mg/100 g of milk), glutamine (2.15 mg/100 g of milk), serine (1.63 mg/100 g) and alanine (1.26 mg/100 g).

On the other hand, the amount of each other protein amino acid did not exceed 5.8 mg/100 g of the product (~12% of free amino acids total content). The analysis also revealed the presence of twelve non-protein amino acids (i.e., L-α-aminoadipic acid (AAAA); L-α-amminobutirrico (ABAA); β-alanine (β-Ala); L-citrulline (Citr); ethanolamine (Ethan); 1-Methylhistidine (1-Mhis); 3-Methylhistidine (3-Mis); L-ornithine (Orn); phosphorylethanolamine (Pea); phosphoserine (Phser); L-sarcosine (Sarc); taurine (Taur)). The amount of these non-protein amino acids was 24.0 mg/100 g of milk (~52% of total). Finally, the analysis revealed that urea content in ‘Alto Casertano’ raw milk (67.88 mg/100 g of milk) represents about 22% of the NPN fraction from ‘Alto Casertano’ goat raw milk, while free amino acids represent about 15% of the NPN fraction.

### 3.5. Amino Acid Content of Cow, Sheep and Goat Raw Milk from ‘Alto Casertano’

Raw milk got from some mammalian species is one of the most important sources of proteins for human nutrition [23]. The total amino acid content (free plus protein) per 100 g of proteins from cow, sheep and goat milk is reported in Table 7. In particular, comparing the total amino acid content among the three species raised in the Ailano and Valle Agricola (Alto Casertano) territories, no qualitative differences were observed among cow, sheep and goat ‘Alto Casertano’ raw milk, while quantitative differences were found. Glx (glutamic acid + glutamine) was by far the most abundant among the total amino acids (about 21.67%, 19.69% and 19.71% for cow, sheep and goat raw milk, respectively). Leucine, lysine and Asx (aspartic acid + asparagine) content was quite abundant (about 7–8%) in the three different species, while the proline content is the most abundant in sheep raw milk (9.31%) with respect to goat and cow raw milk (8.94% and 6.5%, respectively). Furthermore, the content of other amino acids did not exceed 5% of the total protein content; in particular, tyrosine, serine, alanine, arginine, isoleucine and histidine content were higher in cow and sheep raw milk than in goat raw milk.

In addition, the content of valine, threonine and phenylalanine was higher in sheep and goat raw milk than in cow raw milk, while glycine content was the same for the three species. Moreover, the amount of methionine and cysteine in the three species did not exceed the 4% of total protein, confirming the low level of sulfur amino acids. These data show that the quantity of essential amino acids in the three different types of raw milk is about 40% of the total proteins, confirming the good protein quality of this food.

**Figure 3:**
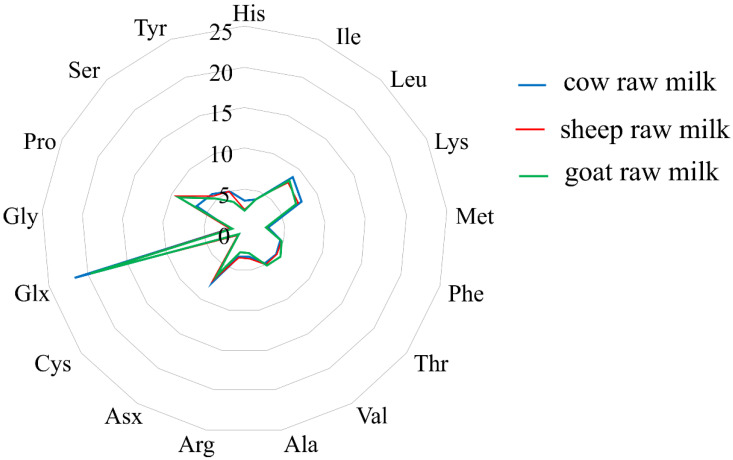
Radar graph of the average raw milk amino acid composition from three different mammal species (cow, sheep and goat), raised in two localities of ‘Alto Casertano’.

The results discussed in this work demonstrate how the amino acid content of the milks analyzed changes among the different species, which is also shown by the radar chart of the average milk amino acid composition from three different mammal species (cow, sheep and goat), raised in two localities of ‘Alto Casertano’ (Campania region, Italy) (Figure 3).

**Figure 4:**
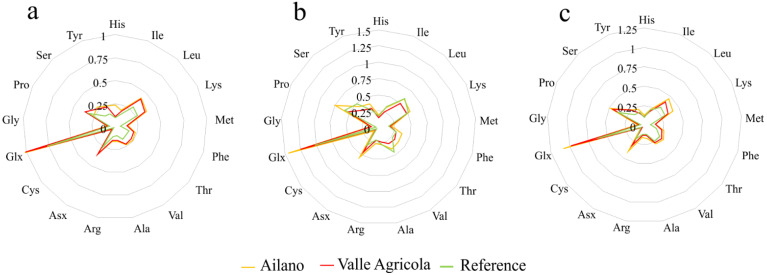
Radar graphs of the total amino acid content from the three types of milk analyzed, sampled in Ailano and Valle Agricola mountain localities, in comparison with the reference values [22]. (**a**) cow raw milk, (**b**) sheep raw milk and (**c**) goat raw milk.

Furthermore, in Figure 4, the total amino acid profiles obtained from raw milks were compared with the milk values reported by Claeys et al., showing that the amino acid profiles of cow, sheep and goat raw milk are similar to those previously reported [22]. On the other hand, the amino acid content of mountain milk (expressed as g/100 g of milk) is higher than that of the milk values reported by Claeys et al. [22].

On the other hand, comparing the free amino acid content among cow, sheep and goat raw milk, raised in the ‘Alto Casertano’, qualitative and quantitative differences were found. The total free amino acid content per 100 g of cow, sheep and goat ‘Alto Casertano’ raw milk was 21.33, 21.86 and 46.15 mg, respectively (Table 8).

Glutamic acid is the most abundant among the free protein amino acids in cow raw milk (~43% of total), while it represents ~14% and ~9% in sheep and goat raw milk, respectively. Vice versa, the most abundant free protein amino acids in sheep and goat raw milk were tyrosine and glycine, respectively. In particular, tyrosine represents about 22% of total free amino acids in sheep raw milk and about 1% in both cow and goat raw milk. Glycine represents about 10% of total free amino acids in goat raw milk and about 7 and 5% of total free amino acids in cow and sheep raw milk, respectively. Furthermore, glutamine is present only in goat raw milk (~5% of total free amino acids), while asparagine and alanine represent about 6% and 3% of total free amino acids in goat raw milk and about 4% and 2% in cow and sheep raw milk, respectively. Moreover, the amount of free essential amino acids, was about 7% of total free amino acids in both cow and goat raw milk and 12% of total free amino acids in sheep raw milk vs. 40% of total amino acids (free plus protein), not significant as a contribution in a human diet.

The analysis also revealed the presence of quali-quantitative differences in non-protein amino acids. In particular, the amount of non-protein amino acids in cow, sheep and goat raw milk was 29%, 34% and 52% of total free amino acids, respectively. In particular, L-α-aminobutyric acid is present only in cow and goat raw milk (~2% and 1% of total non-protein amino acids, respectively), while 1-Methylhistidine and 3-Methylhistidine were present only in sheep and goat raw milk (~1.0% and 0.6% 1-Mhis; ~0.6% and 0.4% 3-Mhis of total non-protein amino acids, respectively). Finally, L-carnitine is present only sheep raw milk (~0.4% of total non-protein amino acids). Furthermore, taurine is an aminosulfonic acid, derived from methionine and cysteine and in strict sense, it is not an amino acid. Taurine is an essential nutrient for the infant due to its insufficient endogenous synthesis. Taurine may act as a membrane stabilizer and growth modulator and plays a role in the formation of bile acids, which facilitates lipid digestion and absorption [24].

The data obtained in this study highlight that goat raw milk is a good source of the amino acid taurine, which represents about 32% of total free amino acids, as previously reported [25]; meanwhile, the content of taurine in cow and sheep raw milk was about 7% and 10%, respectively.

**Figure 5:**
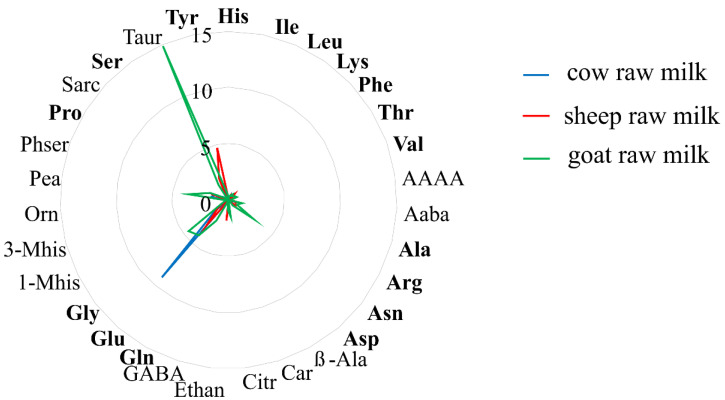
Radar graph of the average free amino acid profile from cow, sheep and goat raw milk analyzed samples.

Finally, as shown in the radar graphs (Figure 5), a comparison of the average free amino acid profile from cow, sheep and goat raw milk analyzed reveals that the free amino acid footprint of the three milk types changes among the species analyzed.

## 4. Conclusions

Consumers associate the value and quality of raw milk with uncontaminated breeding places, such as mountain territories, where the animals are kept in a semi-wild regime. In this scenario, confirming the goodness of raw milk from mountainous areas can be useful to encourage local production and, subsequently, commercialization. Raw milk is a reservoir of high-quality proteins and the best source of nutrition for nearly all infants, containing all nutrients necessary for the growth and development of newborn [26]. Moreover, raw milk from different species (e.g., cow, goat and sheep) continues to be part of human nutrition in adult life. For these reasons, the free and total amino acid profile of milk from different species plays a key role for both milk producers and processors, as well as for consumers, in order to reach innovative product design, versatility, taste and functionality.

In light of this, we investigated the total protein content (caseins plus whey proteins) and total and free amino acid profiles of raw milk from cow, sheep and goat raised in Ailano and Valle Agricola territories, two mountain localities of ‘Alto Casertano’ (Campania region, Italy). In particular, the three raw milk samples analyzed showed higher total amino acid content with respect to milk values reported by Claeys et al. [22]. 

On the other hand, free amino acid profiles from cow, sheep and goat raw milks are characteristic and can be used as a hallmark of these species.

Overall, the higher quality of the three different mountain raw milk analyzed samples from Ailano and Valle Agricola could justify the possibility of the highest retail price of this product and its derivatives, encouraging the local farmers to increase milk production in order to provide adequate incomes for the local small farms, converting their economy from subsistence incomes to profit ones.

## Figures and Tables

**Table 1:** Total amino acid composition of cow raw milk from ‘Ailano’ and ‘Valle Agricola’. Values are means (±SD) and are expressed as g per 100 g of milk. See Section 2.2 of Materials and Methods section for more details.

*Total Amino Acid Composition of Cow Raw Milk*					
Amino Acid	‘Ailano’	‘Valle Agricola’	*p* Value	Average Values	W.L. Claeys et al.
*Essential amino acids*					
His	0.24 ± 0.06 a	0.11 ± 0.00 b	<0.001	0.17	0.10
Ile	0.19 ± 0.02 a	0.18 ± 0.00 a	>0.05	0.19	0.14
Leu	0.42 ± 0.04 a	0.41 ± 0.01 a	>0.05	0.41	0.29
Lys	0.38 ± 0.01 a	0.36 ± 0.01 a	>0.05	0.37	0.27
Met	0.14 ± 0.01 a	0.13 ± 0.00 a	>0.05	0.14	0.06
Phe	0.23 ± 0.02 a	0.21 ± 0.00 a	>0.05	0.22	0.16
Thr	0.24 ± 0.02 a	0.22 ± 0.00 a	>0.05	0.23	0.15
Trp	n.d.	n.d.	-	-	0.05
Val	0.23 ± 0.02 a	0.22 ± 0.00 a	>0.05	0.23	0.16
*Non-essential amino acids*					
Ala	0.16 ± 0.01 a	0.15 ± 0.00 a	>0.05	0.16	0.10
Arg	0.16 ± 0.02 a	0.15 ± 0.00 a	>0.05	0.16	0.11
Asx	0.35 ± 0.03 a	0.35 ± 0.01 a	>0.05	0.35	0.26
Cys ^&^	0.04 ± 0.00 a	0.04 ± 0.00 a	>0.05	0.04	0.02
Glx	1.02 ± 0.06 a	1.01 ± 0.02 a	>0.05	1.02	0.77
Gly	0.09 ± 0.01 a	0.08 ± 0.00 a	>0.05	0.08	0.06
Pro	0.26 ± 0.03 a	0.36 ± 0.00 b	<0.001	0.31	0.32
Ser	0.29 ± 0.03 a	0.27 ± 0.01 a	>0.05	0.28	0.16
Tyr	0.24 ± 0.02 a	0.23 ± 0.00 a	>0.05	0.23	0.15
**Total**	**4.69**	**4.48**		**4.58**	**3.33**

For protein amino acids, a three-letter code has been used. ^&^ Cys amount was evaluated after performic acid oxidation. n.d., not determined. Values followed by different letters within a row are significantly (*p* < 0.05) different by Bonferroni post-test.

**Table 2:** Free amino acid composition of cow raw milk from ‘Ailano’ and ‘Valle Agricola’. Values are means (±SD) and are expressed as mg per 100 g of milk. See Section 2.2 of Materials and Methods section for more details.

*Free Amino Acid Composition of Cow Raw Milk*				
Amino Acid ^#^	Ailano	Valle Agricola	*p* Value	Average Values
*Essential amino acids*				
**His**	0.06 ± 0.01 a	0.05 ± 0.01 a	>0.05	0.06
**Ile**	0.14 ± 0.01 a	0.25 ± 0.01 a	>0.05	0.20
**Leu**	0.14 ± 0.00 a	0.23 ± 0.01 a	>0.05	0.19
**Lys**	0.53 ± 0.07 a	0.28 ± 0.04 a	>0.05	0.41
**Met**	n.d.	n.d.	-	-
**Phe**	0.24 ± 0.00 a	0.24 ± 0.01 a	>0.05	0.24
**Thr**	0.17 ± 0.08 a	0.16 ± 0.00 a	>0.05	0.17
**Trp**	n.d.	n.d.	-	-
**Val**	0.21 ± 0.00 a	0.20 ± 0.01 a	>0.05	0.21
*Non-essential amino acids*				
AAAA	0.13 ± 0.00 a	0.12 ± 0.01 a	>0.05	0.13
ABAA	0.15 ± 0.02 a	0.10 ± 0.02 a	>0.05	0.13
**Ala**	1.01 ± 0.12 a	0.80 ± 0.00 a	>0.05	0.91
**Arg**	0.40 ± 0.03 a	0.27 ± 0.06 a	>0.05	0.34
**Asn**	0.33 ± 0.02 a	0.23 ± 0.02 a	>0.05	0.28
**Asp**	0.68 ± 0.04 a	0.71 ± 0.00 a	>0.05	0.70
β-Ala	0.11 ± 0.02 a	0.14 ± 0.01 a	>0.05	0.13
Car	n.d	n.d.	-	-
Citr	0.17 ± 0.01 a	0.11 ± 0.01 a	>0.05	0.14
**Cys**	n.d.	n.d	-	-
Ethan	1.49 ± 0.30 a	0.82 ± 0.14 a	>0.05	1.16
GABA	n.d.	0.02 ± 0.00	>0.05	-
**Gln**	n.d.	n.d.	-	-
**Glu**	6.18 ± 0.21 a	11.96 ± 0.18 b	<0.001	9.07
**Gly**	1.39 ± 0.05 a	1.65 ± 0.03 a	>0.05	1.52
Homocys	n.d.	n.d.	-	-
1-Mhis	n.d	n.d.	-	-
3-Mhis	n.d.	n.d.	-	-
Orn	0.06 ± 0.00 a	0.09 ± 0.00 a	>0.05	0.08
Pea	1.19 ± 0.07 a	1.94 ± 0.08 a	>0.05	1.57
Phser	1.34 ± 0.03 a	1.36 ± 0.01 a	>0.05	1.35
**Pro**	0.58 ± 0.08 a	0.37 ± 0.03 a	>0.05	0.48
Sarc	0.13 ± 0.00 a	0.15 ± 0.01 a	>0.05	0.14
**Ser**	0.30 ± 0.02 a	0.30 ± 0.04 a	>0.05	0.30
Taur	1.39 ± 0.02 a	1.37 ± 0.05 a	>0.05	1.38
**Tyr**	0.18 ± 0.06 a	0.03 ± 0.01 a	>0.05	0.11
**Total**	**18.70**	**23.95**	-	**21.40**
*Urea*	*46.58 ± 1.00* a	*41.16 ± 3.24* b	<0.001	*43.87*

^#^ Free and protein amino acids. n.d., not detected. Three-letter code has been used. Values followed by different letters within a row are significantly (*p* < 0.05) different by Bonferroni post-test. Protein amino acids are highlighted in bold.

**Table 3:** Total amino acid composition of sheep raw milk from ‘Ailano’ and ‘Valle Agricola’. Values are means (±SD) and are expressed as g per 100 g of milk. See Section 2.2 of Materials and Methods section for more details.

*Total Amino Acid Composition of Sheep Raw Milk*					
Amino Acid	‘Ailano’	‘Valle Agricola’	*p* Value	Average Values	W.L. Claeys et al.
*Essential amino acids*					
His	0.19 ± 0.00 a	0.15 ± 0.01 a	>0.05	0.17	0.17
Ile	0.32 ± 0.00 a	0.25 ± 0.01 b	<0.01	0.29	0.34
Leu	0.58 ± 0.00 a	0.50 ± 0.02 b	<0.01	0.54	0.59
Lys	0.54 ± 0.00 a	0.48 ± 0.06 b	<0.05	0.51	0.51
Met	0.21 ± 0.00 a	0.18 ± 0.01 a	>0.05	0.19	0.16
Phe	0.37 ± 0.00 a	0.28 ± 0.01 b	<0.001	0.33	0.28
Thr	0.38 ± 0.01 a	0.31 ± 0.01 b	<0.01	0.35	0.27
Trp	n.d.	n.d.	-	-	0.08
Val	0.39 ± 0.00 a	0.30 ± 0.01 b	<0.001	0.34	0.45
*Non-essential amino acids*					
Ala	0.27 ± 0.00 a	0.23 ± 0.02 a	>0.05	0.25	0.27
Arg	0.26 ± 0.00 a	0.21 ± 0.01 a	>0.05	0.23	0.20
Asx	0.55 ± 0.00 a	0.45 ± 0.03 b	<0.001	0.50	0.33
Cys ^&^	0.09 ± 0.01 a	0.05 ± 0.01 a	>0.05	0.07	0.04
Glx	1.45 ± 0.00 a	1.25 ± 0.10 b	<0.001	1.35	1.02
Gly	0.13 ± 0.00 a	0.11 ± 0.01 a	>0.05	0.12	0.04
Pro	0.74 ± 0.00 a	0.53 ± 0.03 b	<0.001	0.64	0.58
Ser	0.43 ± 0.01 a	0.35 ± 0.02 b	<0.01	0.39	0.49
Tyr	0.38 ± 0.00 a	0.31 ± 0.01 b	<0.01	0.34	0.28
**Total**	**7.30**	**5.93**	**-**	**6.62**	**6.09**

For protein amino acids, a three-letter code has been used. ^&^ Cys amount was evaluated after performic acid oxidation. n.d., not detected. Values followed by different letters within a row are significantly (*p* < 0.05) different by Bonferroni post-test.

**Table 4:** Free amino acid composition of sheep raw milk from ‘Ailano’ and ‘Valle Agricola’. Values are means (±SD) and are expressed as mg per 100 g of milk. See Section 2.2 of Materials and Methods section for more details.

*Free Amino Acid Composition of Sheep Raw Milk*				
Amino Acid ^#^	Ailano	Valle Agricola	*p* Value	Average Values
*Essential amino acids*				
**His**	0.09 ± 0.00 a	0.13 ± 0.01 a	>0.05	0.11
**Ile**	0.21 ± 0.01 a	0.23 ± 0.03 a	>0.05	0.22
**Leu**	0.56 ± 0.05 a	0.30 ± 0.03 a	>0.05	0.43
**Lys**	0.29 ± 0.00 a	0.24 ± 0.01 a	>0.05	0.27
**Met**	0.03 ± 0.00	n.d.	>0.05	-
**Phe**	1.11 ± 0.03 a	0.35 ± 0.01 a	>0.05	0.73
**Thr**	0.64 ± 0.02 a	0.28 ± 0.00 a	>0.05	0.46
**Trp**	n.d.	n.d.	-	0.00
**Val**	0.47 ± 0.00 a	0.36 ± 0.00 a	>0.05	0.42
*Non-essential amino acids*				
AAAA	0.14 ± 0.00 a	0.10 ± 0.02 a	>0.05	0.12
ABAA	0.06 ± 0.01	n.d.	>0.05	-
**Ala**	0.81 ± 0.00 a	0.39 ± 0.00 a	>0.05	0.60
**Arg**	0.78 ± 0.03 a	0.63 ± 0.01 a	>0.05	0.71
**Asn**	0.60 ± 0.13 a	0.93 ± 0.04 a	>0.05	0.77
**Asp**	0.39 ± 0.01 a	0.27 ± 0.01 a	>0.05	0.33
β-Ala	0.25 ± 0.03 a	0.13 ± 0.02 a	>0.05	0.19
Car	0.08 ± 0.01 a	0.09 ± 0.00 a	>0.05	0.09
Citr	0.55 ± 0.01 a	0.39 ± 0.05 a	>0.05	0.47
**Cys**	n.d.	n.d.	-	-
Ethan	2.03 ± 0.00 a	1.62 ± 0.12 a	>0.05	1.83
GABA	0.02 ± 0.01	n.d.	>0.05	0.01
**Gln**	n.d.	n.d.	-	-
**Glu**	4.08 ± 0.07 a	1.87 ± 0.04 b	<0.001	2.98
**Gly**	1.35 ± 0.02 a	0.89 ± 0.08 a	>0.05	1.12
Homocys	0.03 ± 0.01	n.d.	>0.05	-
1-Mhis	0.21 ± 0.00 a	0.40 ± 0.03 a	>0.05	0.31
3-Mhis	0.09 ± 0.04 a	0.19 ± 0.02 a	>0.05	0.14
Orn	0.15 ± 0.00 a	0.14 ± 0.00 a	>0.05	0.15
Pea	0.50 ± 0.01 a	0.08 ± 0.02 a	>0.05	0.29
Phser	1.90 ± 0.03 a	1.35 ± 0.07 a	>0.05	1.63
**Pro**	0.43 ± 0.02 a	0.13 ± 0.02 a	>0.05	0.28
Sarc	0.15 ± 0.03 a	0.14 ± 0.00 a	>0.05	0.15
**Ser**	0.31 ± 0.06 a	0.25 ± 0.02 a	>0.05	0.28
Taur	0.42 ± 0.02 a	3.78 ± 0.11 b	<0.001	2.10
**Tyr**	9.12 ± 0.09 a	0.32 ± 0.05 b	<0.001	4.72
**Total**	**27.85**	**15.98**	**-**	**21.86**
*Urea*	*81.30 ± 1.80* a	*37.90 ± 1.61* b	<0.001	*59.6*

^#^ Free and protein amino acids. n.d., not detected. Three-letter code has been used. Values followed by different letters within a row are significantly (*p* < 0.05) different by Bonferroni post-test. Protein amino acids are highlighted in bold.

**Table 5:** Total amino acid composition of goat raw milk from ‘Ailano’ and ‘Valle Agricola’. Values are means (±SD) and are expressed as g per 100 g of milk. See Section 2.2 of Materials and Methods section for more details.

*Total Amino Acid Composition of Goat Raw Milk*					
Amino Acid	‘Ailano’	‘Valle Agricola’	*p* Value	Average Values	W.L. Claeys et al.
*Essential amino acids*					
His	0.13 ± 0.02 a	0.11 ± 0.00 a	>0.05	0.12	0.10
Ile	0.22 ± 0.03 a	0.19 ± 0.01 a	>0.05	0.21	0.21
Leu	0.46 ± 0.03 a	0.40 ± 0.02 a	>0.05	0.43	0.31
Lys	0.41 ± 0.03 a	0.34 ± 0.01 b	<0.05	0.37	0.29
Met	0.14 ± 0.01 a	0.14 ± 0.01 a	>0.05	0.14	0.08
Phe	0.26 ± 0.03 a	0.23 ± 0.01 a	>0.05	0.25	0.16
Thr	0.30 ± 0.03 a	0.28 ± 0.01 a	>0.05	0.29	0.24
Trp	n.d.	n.d.	-	-	0.04
Val	0.28 ± 0.03 a	0.26 ± 0.01 a	>0.05	0.27	0.24
*Non-essential amino acids*					
Ala	0.17 ± 0.03	0.13 ± 0.01	>0.05	0.15	0.12
Arg	0.16 ± 0.02	0.13 ± 0.01	>0.05	0.14	0.12
Asx	0.39 ± 0.03	0.30 ± 0.01	<0.01	0.34	0.21
Cys ^&^	0.05 ± 0.01	0.04 ± 0.00	>0.05	0.05	0.05
Glx	1.08 ± 0.09	0.98 ± 0.03	<0.001	1.03	0.63
Gly	0.09 ± 0.01	0.07 ± 0.00	>0.05	0.08	0.05
Pro	0.49 ± 0.05	0.45 ± 0.03	>0.05	0.47	0.37
Ser	0.27 ± 0.04	0.27 ± 0.01	>0.05	0.27	0.18
Tyr	0.22 ± 0.02	0.17 ± 0.01	>0.05	0.20	0.18
**Total**	**5.13**	**4.48**	**-**	**4.81**	**3.57**

For protein amino acids, a three-letter code has been used. ^&^ Cys amount was evaluated after performic acid oxidation. n.d. not determined. Values followed by different letters within a row are significantly (*p* < 0.05) different by Bonferroni post-test.

**Table 6:** Free amino acid composition of goat raw milk from ‘Ailano’ and ‘Valle Agricola’. Values are means (±SD) and are expressed as mg per 100 g of milk. See Section 2.2 of Materials and Methods section for more details.

*Free Amino Acid Composition of Goat Raw Milk*				
Amino acid ^#^	Ailano	Valle Agricola	*p* Value	Average Values
*Essential amino acids*				
**His**	0.05 ± 0.00 a	0.16 ± 0.01 a	>0.05	0.11
**Ile**	0.19 ± 0.01 a	0.37 ± 0.04 a	>0.05	0.28
**Leu**	0.25 ± 0.10 a	0.67 ± 0.07 a	>0.05	0.46
**Lys**	0.51 ± 0.02 a	0.78 ± 0.00 a	>0.05	0.65
**Met**	n.d.	n.d.	-	-
**Phe**	0.39 ± 0.01 a	0.49 ± 0.01 a	>0.05	0.44
**Thr**	0.84 ± 0.01 a	0.54 ± 0.03 a	>0.05	0.69
**Trp**	n.d.	n.d	-	-
**Val**	0.63 ± 0.01 a	0.98 ± 0.01 a	>0.05	0.81
*Non-essential amino acids*				
AAAA	0.08 ± 0.01 a	0.14 ± 0.05 a	>0.05	0.11
ABAA	0.31 ± 0.00 a	0.10 ± 0.01 a	>0.05	0.21
**Ala**	1.33 ± 0.04 a	1.19 ± 0.02 a	>0.05	1.26
**Arg**	0.48 ± 0.02 a	1.24 ± 0.03 a	>0.05	0.86
**Asn**	1.89 ± 0.43 a	3.60 ± 0.02 a	>0.05	2.75
**Asp**	0.87 ± 0.00 a	0.33 ± 0.02 a	>0.05	0.60
β-Ala	0.18 ± 0.00 a	0.07 ± 0.00 a	>0.05	0.13
Car	n.d.	n.d.	-	-
Citr	1.67 ± 0.04 a	1.39 ± 0.04 a	>0.05	1.53
**Cys**	n.d.	0.07 ± 0.04	>0.05	-
Ethan	0.78 ± 0.14 a	0.95 ± 0.01 a	>0.05	0.87
GABA	n.d.	0.02 ± 0.00	>0.05	-
**Gln**	1.55 ± 0.22 a	2.74 ± 0.08 a	>0.05	2.15
**Glu**	3.36 ± 0.01 a	4.88 ± 0.04 a	>0.05	4.12
**Gly**	4.29 ± 0.00 a	4.78 ± 0.03 a	>0.05	4.54
Homocys	n.d.	n.d.	-	-
1-Mhis	0.34 ± 0.00 a	0.38 ± 0.04 a	>0.05	0.36
3-Mhis	0.18 ± 0.01 a	0.20 ± 0.02 a	>0.05	0.19
Orn	0.14 ± 0.00 a	0.28 ± 0.01 a	>0.05	0.21
Pea	4.15 ± 0.07 a	2.99 ± 0.10 a	>0.05	3.57
Phser	1.48 ± 0.04 a	2.04 ± 0.13 a	>0.05	1.76
**Pro**	0.16 ± 0.01 a	0.44 ± 0.02 a	>0.05	0.30
Sarc	0.11 ± 0.01 a	0.17 ± 0.03 a	>0.05	0.14
**Ser**	1.59 ± 0.00 a	1.66 ± 0.06 a	>0.05	1.63
Taur	16.53 ± 0.00 a	13.30 ± 0.05 a	<0.05	14.92
**Tyr**	0.47 ± 0.00 a	0.64 ± 0.10 a	>0.05	0.56
**Total**	**44.8**	**47.59**	**-**	**46.15**
*Urea*	*53.44 ± 5.04*	*82.32 ± 8.32*	<0.001	*67.88*

^#^ Free and protein amino acids. n.d., not detected. Three-letter code has been used. Values followed by different letters within a row are significantly (*p* < 0.05) different by Bonferroni post-test. Protein amino acids are highlighted in bold.

**Table 7:** Total amino acid composition of ‘Alto Casertano’ raw milk samples. Values are means from ‘Ailano’ and ‘Valle Agricola’ milk and are expressed as g per 100 g of total protein.

*Total Amino Acid Composition of Alto Casertano Milk*			
Amino Acid	Cow	Sheep	Goat
*Essential amino acids*			
His	3.66	2.52	2.34
Ile	4.01	4.22	3.93
Leu	8.81	7.91	8.23
Lys	7.79	7.47	7.15
Met	2.91	2.80	2.65
Phe	4.67	4.76	4.75
Thr	4.87	5.06	5.47
Trp	n.d.	n.d.	n.d.
Val	4.79	5.01	5.22
*Non-essential amino acids*			
Ala	3.31	3.64	2.92
Arg	3.33	3.40	2.75
Asx	7.49	7.27	6.55
Cys	0.87	1.06	0.87
Glx	21.67	19.69	19.71
Gly	1.80	1.74	1.52
Pro	6.58	9.31	8.94
Ser	5.96	5.69	5.16
Tyr	5.00	5.04	3.78

For protein amino acids, a three-letter code has been used. n.d., not determined.

**Table 8:** Free amino acid composition of ‘Alto Casertano’ milk samples. Values are means from ‘Ailano’ and ‘Valle Agricola’ milk and are expressed as mg per 100 g of milk.

*Free Amino Acid Composition of ‘Alto Casertano’ Milk*			
Amino Acid ^a^	Cow	Sheep	Goat
*Essential amino acids*			
**His**	0.06	0.11	0.11
**Ile**	0.20	0.22	0.28
**Leu**	0.19	0.43	0.46
**Lys**	0.41	0.27	0.65
**Met**	-	-	-
**Phe**	0.24	0.73	0.44
**Thr**	0.17	0.46	0.69
**Trp**	-	0.00	-
**Val**	0.21	0.42	0.81
*Non-essential amino acids*			
AAAA	0.13	0.12	0.11
Aaba	0.13	-	0.21
**Ala**	0.91	0.60	1.26
**Arg**	0.34	0.71	0.86
**Asn**	0.28	0.77	2.75
**Asp**	0.70	0.33	0.60
β-Ala	0.13	0.19	0.13
Car	-	0.09	-
Citr	0.14	0.47	1.53
**Cys**	-	-	-
Ethan	1.16	1.83	0.87
GABA	-	0.01	-
**Gln**	-	-	2.15
**Glu**	9.07	2.98	4.12
**Gly**	1.52	1.12	4.54
Homocys	-	-	-
1-Mhis	-	0.31	0.36
3-Mhis	-	0.14	0.19
Orn	0.08	0.15	0.21
Pea	1.57	0.29	3.57
Phser	1.35	1.63	1.76
**Pro**	0.48	0.28	0.30
Sarc	0.14	0.15	0.14
**Ser**	0.30	0.28	1.63
Taur	1.38	2.10	14.92
**Tyr**	0.11	4.72	0.56
**Total**	**21.33**	**21.86**	**46.15**

^a^, free and protein amino acids. Three-letter code has been used. Protein amino acids are highlighted in bold.

## Data Availability

Raw data can be provided by the corresponding author on request.

## Abbreviations

For the amino acids, the standard tree-letter code has been used.
